# Biotechnological Preparedness for Novel Pandemics: Diagnostic Performance of IVDS Against SARS‐CoV‐2

**DOI:** 10.1002/mbo3.70042

**Published:** 2025-08-04

**Authors:** Murat Kavruk, Meltem Ercan, Barış Ata Borsa, Veli Cengiz Özalp, Frank J. Hernandez

**Affiliations:** ^1^ Department of Medical Biology and Genetics, Faculty of Medicine, Department of Molecular Biology and Genetics, Faculty of Arts & Sciences İstanbul Aydın University İstanbul Türkiye; ^2^ Department of Physics, Chemistry and Biology (IFM) Linköping University Linköping Sweden; ^3^ Deparment of Medical Biology, Faculty of Medicine Atılım University Ankara Türkiye; ^4^ Department of Bioengineering and Biosciences, TECNUN Navarra University Spain; ^5^ IKERBASQUE, Basque Foundation for Science Bilbao Spain

**Keywords:** COVID, disease X, IVD kits, public health, SARS‐CoV‐2

## Abstract

Although the COVID‐19 pandemic has created many challenges and negative impacts around the world, some of which will persist into the future, its technological challenge has created a unique opportunity in a globalized world. It is a rare event that almost all of humanity to be directed towards a single goal and to try to produce solutions, but the necessity of a similar global action in the future has begun to enter the agenda again. The predictions made on the basis of countries and institutions against the possibility of a pandemic, which is defined as Disease X, are shaped by the experience of the COVID‐19 pandemic. Technologically, one of the know‐how we have gained in this pandemic is the performance of IVD and test systems in terms of quality and quantity. A comprehensive analysis of the products produced by combining biotechnology with different strategies has not been conducted. In this context, we have analyzed the technical preferences, limitations, and other performance parameters of IVDs and test kits that could be developed against a future Disease X. The performance parameters of 2,882 biotechnological products listed for use in the European Union have been analyzed, and areas that could be targeted for increased effectiveness have been identified. Our study is the first of its kind in this field and can serve as a guide for those who want to work on detection methods, diagnostics, and novel technologies for deployment in future pandemics.

## Graphical abstract

During the COVID‐19 pandemic, biotechnological IVDs for SARS‐CoV‐2 detection advanced rapidly, with nucleic acid tests like RT‐PCR, immuno‐antigen tests for rapid protein detection, and immuno‐antibody tests for tracking immune responses. These methods highlight the crucial role of diagnostics in managing pandemics.

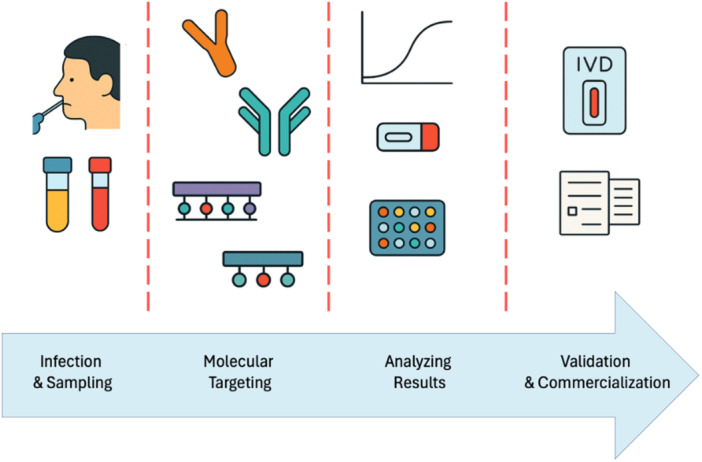

## 1 Introduction

With the World Health Organization declaring it a pandemic on March 11, 2020, SARS‐CoV‐2 changed the world's agenda in a way that continues until today (WHO 2020). Although the impact of the pandemic has decreased, it created perhaps irreversible changes in pre‐pandemic systems, practices, research, and ways of doing business. Due to the scale of these effects, risk analyses have begun on precautions and what to do about a new epidemic disease defined as “Disease X” in different segments of society, from the World Health Organization (WHO 2024) to the World Economic Forum (WEF 2024). Considering that the work done and to be done on this subject will be shaped by the experience of the COVID‐19 pandemic, it would be useful to first analyze the point reached from 2020 to the present.

With the onset of the pandemic, research and innovations focused on four areas: treatment of the patient, effective drug discovery, vaccine development, and diagnostic systems production (Shih et al. 2020). With the acceptance that isolation and diagnosis are the best practices that can be implemented to control the epidemic at a level that the health systems of countries can manage until drug and vaccine development studies are completed (Peck 2020), there has been a race in societies; to take lockdown measures and in technology companies; to develop fast and reliable diagnostic kits. In terms of detection methods, which is also the focus of this study, with the publication of the Wuhan‐1 whole genome sequence (Wu et al. 2020), all methods in the scientific and technological arsenal have been directed towards this goal (Pouresmaieli et al. 2021), from traditional PCR to innovative ones such as CRISPR/Cas (Lucia et al. 2020) or AI support (Yao et al. 2020).

Although computed tomography (CT) and reverse transcriptase polymerase chain reaction (RT‐PCR) methods are considered complementary and sufficient to detect the SARS‐CoV‐2 virus and the pulmonary symptoms of the COVID‐19 pandemic caused by it (Long et al. 2020), alternatives to hospital‐based CT and RT‐PCR methods are needed for millions of detection studies worldwide, as these methods face speed challenges due to the triad of collection, transportation, and analysis of samples (Verma et al. 2021). Among the three different COVID‐19 tests; molecular tests, antigen tests and antibody tests (FDA 2023b) scientific and technological efforts were focused on these types with variations in platfom, detection technology, and even sampling (swab, saliva or blood) for optimization between limit of detection and testing time according to urgent pandemic‐era expectations (Borges et al. 2021; Dinnes et al. 2022).

**Figure 1:**
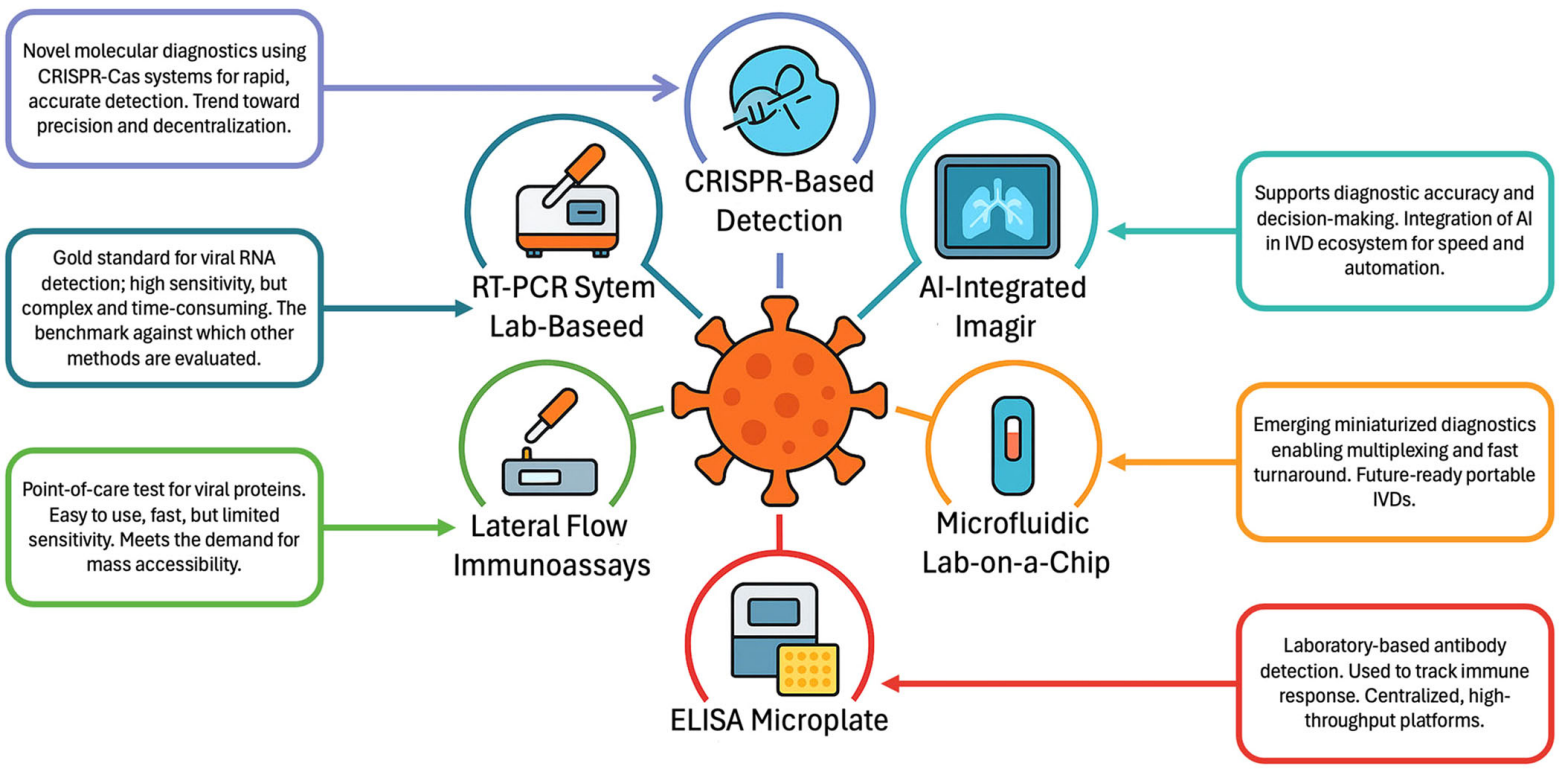
Technological spectrum of diagnostics for SARS‐CoV‐2.

The pandemic catalyzed the rapid development and deployment of decentralized, point‐of‐care (POC) testing with new and innovative technological improvements (Huang et al. 2023; Tuna et al. 2022). Efforts to achieve accurate diagnosis have involved various technological strategies, summarized in Figure 1. Initially reliant on laboratory‐based RT‐PCR, the field expanded to include isothermal amplification methods such as RT‐LAMP and RPA, which offered faster results and greater portability. CRISPR‐based diagnostics also gained attention for their ultra‐specific nucleic acid detection capabilities (Kang et al. 2022). Several molecular and antigen‐based tests, such as the Cue COVID‐19 Test and Abbott's ID NOW platform, received Emergency Use Authorization (EUA) and enabled testing outside traditional lab settings, including at‐home use (Valera et al. 2021). In terms of commercialization, there are some regional differences. In China, the regulatory response was more conservative, prioritizing PCR‐based kits, although over 60% of their approved IVDs were for home use. Meanwhile, the U.S. FDA and European regulators rapidly authorized hundreds of tests, including multiplex assays and saliva‐based diagnostics (Lu et al. 2025). These shifts reflect a broader strategy of the global scientific community: to reduce diagnostic bottlenecks, increase accessibility, and prepare for future pandemics by streamlining regulatory pathways and investing in innovative, rapid, and scalable testing platforms.

There are valuable studies in the literature showing the diversity in detection methods, technological features and detection capabilities against SARS‐CoV‐2 (Pouresmaieli et al. 2021; Zhang 2023), however no analysis has been found focusing on the method, technology, duration, and detection capability of commercial detection kits, transfered from bench to field use and utilized for technical verification from treatment to quarantine according to the results. To contribute to this paucity of information in the literature to express the biotechnological strategies against COVID‐19 and visualize the readiness against Disease X; the COVID‐19 detection methods and kits whose information was concatenated by the European Union were analyzed within terms of focusing countries, certification status, detection‐sample‐target interaction, detection time and detection limit distributions.

## 2 Materials and Methods

To conduct this study, two different data sets were used for analysis. The Web of Science (WoS) database was analyzed to reveal the COVID‐19 detection and diagnosis attempts in scientific literature. The results obtained from this database were compiled based on the parameters: country, WoS categories (for the selection of the category in the field of health), and citation topics meso and micro (an algorithm developed by Clarivate InCites that determines the topic addressed through the dynamic and mutual citations of articles) (Clarivate 2021).

The second and the main database examined is the “JRC COVID‐19 IVD Devices and Test Methods” data set, which is made publicly available on the European Union's data.europa.eu website (JRC 2021). In total, information for 2882 COVID‐19 IVD devices and test kits, commercially produced by 1257 different companies and indexed in the European Union database, were obtained. There are 34 different parameters for each commercial product; 19 of these were chosen to be eligible in terms of the data quality to be the subject of our analysis. In nearly all over the world, IVDs and test kits were approved for their national/regional usage. For instance, the U.S. Food and Drug Administration (FDA) issued over 400 Emergency Use Authorizations (EUAs) for COVID‐19 diagnostics during the pandemic, mainly focusing on molecular tests and at‐home collection kits (FDA 2025; Moshkovits and Shepshelovich 2022). In contrast, countries in Asia, particularly South Korea and China, prioritized centralized screening systems and rapid antigen‐based point‐of‐care testing to manage large‐scale outbreaks (Lee and Lee 2020; Park et al. 2021; Wan et al. 2022). However, no technical and/or scientific data are available from the IVDs approved in these regions to compare with the EU data. Thus, this study mainly focuses on the data of the EU due to its scientific depth and availability of information for the commercial products.

Concatenation, tabulation, geographical, and graphical data analysis were performed in Power BI version 2.120 (Microsoft, Redmond, WA, USA) with Sankey 3.1.2 visualization patch and other conventional graphics visualization programs.

## 3 Results and Discussion

**Figure 2:**
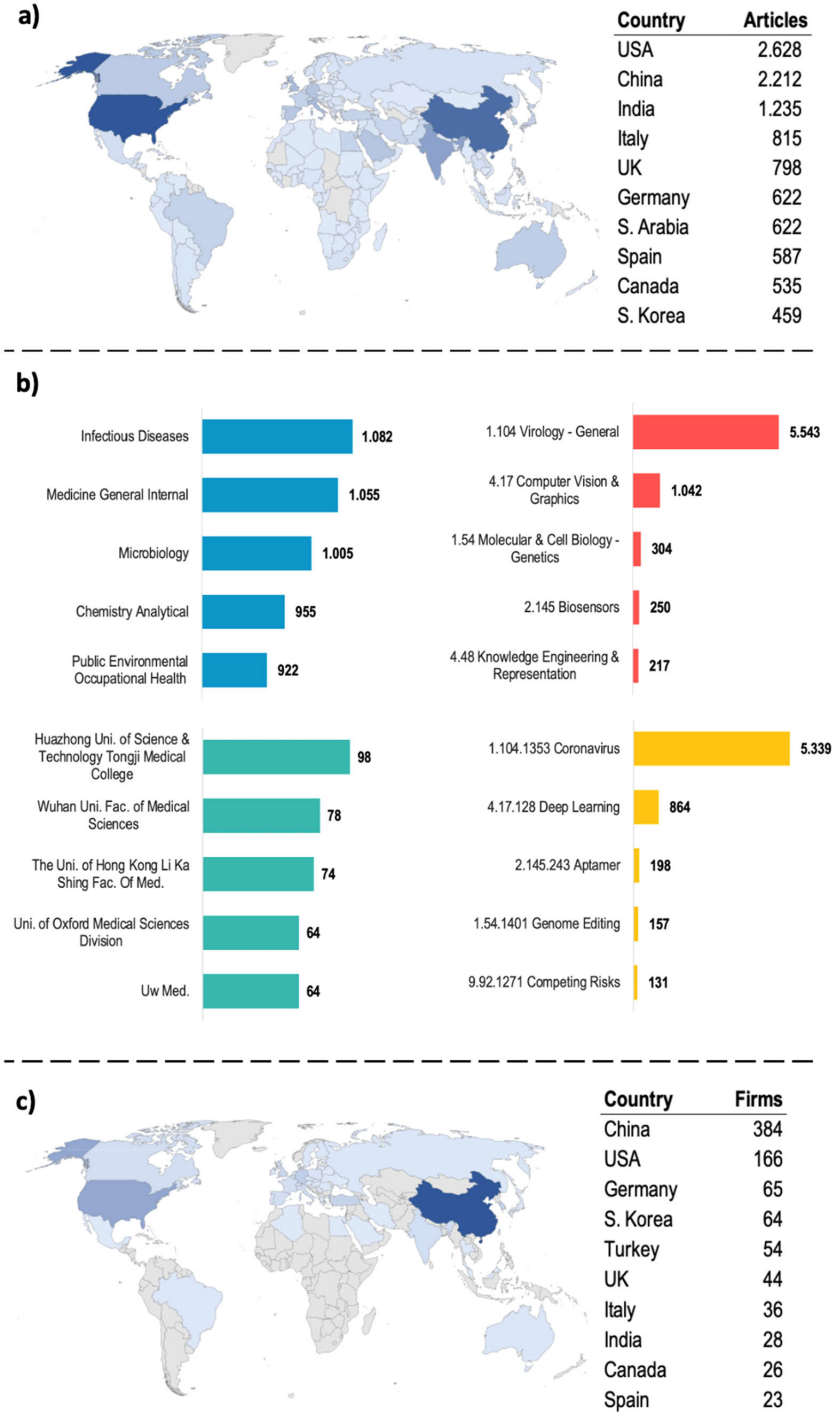
Spatial and topical distribution of COVID‐19 academic studies and technology firms. (a) Number of research articles about COVID‐19 detection in terms of country. (b) Distribution of research articles in terms of WoS category (blue bars), meso citation topics (red bars), micro citation topics (yellow bars), and centers of highest research article numbers (green bars). (c) Geographical distribution of firms producing the IVDs and test kits listed in the European Union database.

Initially, 12,886 research articles were identified as a result of WoS search with the keywords “covid‐19” and “detection”, published simultaneously with the test technologies and kits developed/used commercially during the pandemic. The details of these studies published between 2020 and 2023 are summarized in Figure 2, and when the number of research articles on a country basis is examined, it is observed that the United States and China, as the primary developers of diagnostic kits, differ from other countries (Figure 2a). It has also been revealed in other studies that these two countries share similar leadership in areas that can be defined as scientific and technological hot topics (Kavruk et al. 2024). When the details of the articles are examined using WoS analysis modules, the first five fields as WoS categories are listed according to the number of articles in Figure 2b (blue bars). When the titles Meso Citation Topics (Figure 2b, red bars) and Micro Citation Topics (Figure 2b, yellow bars) created by Clarivate – where the articles are positioned based on their citations – are examined, the titles “2.145 Biosensors” and “2.145.243 Aptamers” have been noted among the top five. The fact that biosensors are to be used in the field as the PCR alternative (considered to be the gold standard) and aptamer technology (aiming to create the “recognition molecule”) has come to the fore, revealing the scientific quest for alternatives to existing technological opportunities. In the centers where the most publications are made (Figure 2b, green bars), the first three Chinese health centers are followed by one center each from England and the USA.

The abundance of publications based in China can be explained by the number of researchers, technological opportunities and general acceptance as the center of origin of the disease. This type of distribution becomes more striking in the country‐based distribution of commercial IVD devices and test kits in the second database we examined in our study (Figure 2c). As a result of primarily meeting domestic demand and then an export‐oriented approach, the number of China‐based IVD device and test kit manufacturing companies is higher than the total number of companies in the following four countries. A total of 2,882 IVD devices and kits in the database are produced in 51 different countries (the origin of 28 kits is not specified). It was determined that the CE approval of the IVD devices and kits examined was 86%. Among the top 10 countries that produce the most different IVDs, the highest CE rate is in Italy (95%), Türkiye (94%), and Spain (92%), while the lowest CE rate is in the USA (53%), India (79%) and UK (84%) originated products (data not shown). No country in Europe that stands out in terms of developing COVID‐19‐focused IVD devices and test kits could be identified.

In light of these data examined, it can be concluded that the USA and China may come to the fore in terms of scientific struggle and technological innovation against Disease X, which poses a serious risk parameter in the near future, while Europe may be far from such an advantage at this stage in terms of commercialization. It is foreseeable that these two countries will come to the fore due to technological initiative and cost‐oriented mass production, respectively. But this picture emerged at the end of a capitalist era without borders. The expectation of meeting the need for affordable kits on a global scale in a short time was a struggle even in the environment during the COVID‐19 period. In the event of a new pandemic in the dynamism of leaving the place of global collaborations to global competition and regional polarizations, having and/or having access to basic kit components will significantly determine the fight against the pandemic of societies.

**Figure 3:**
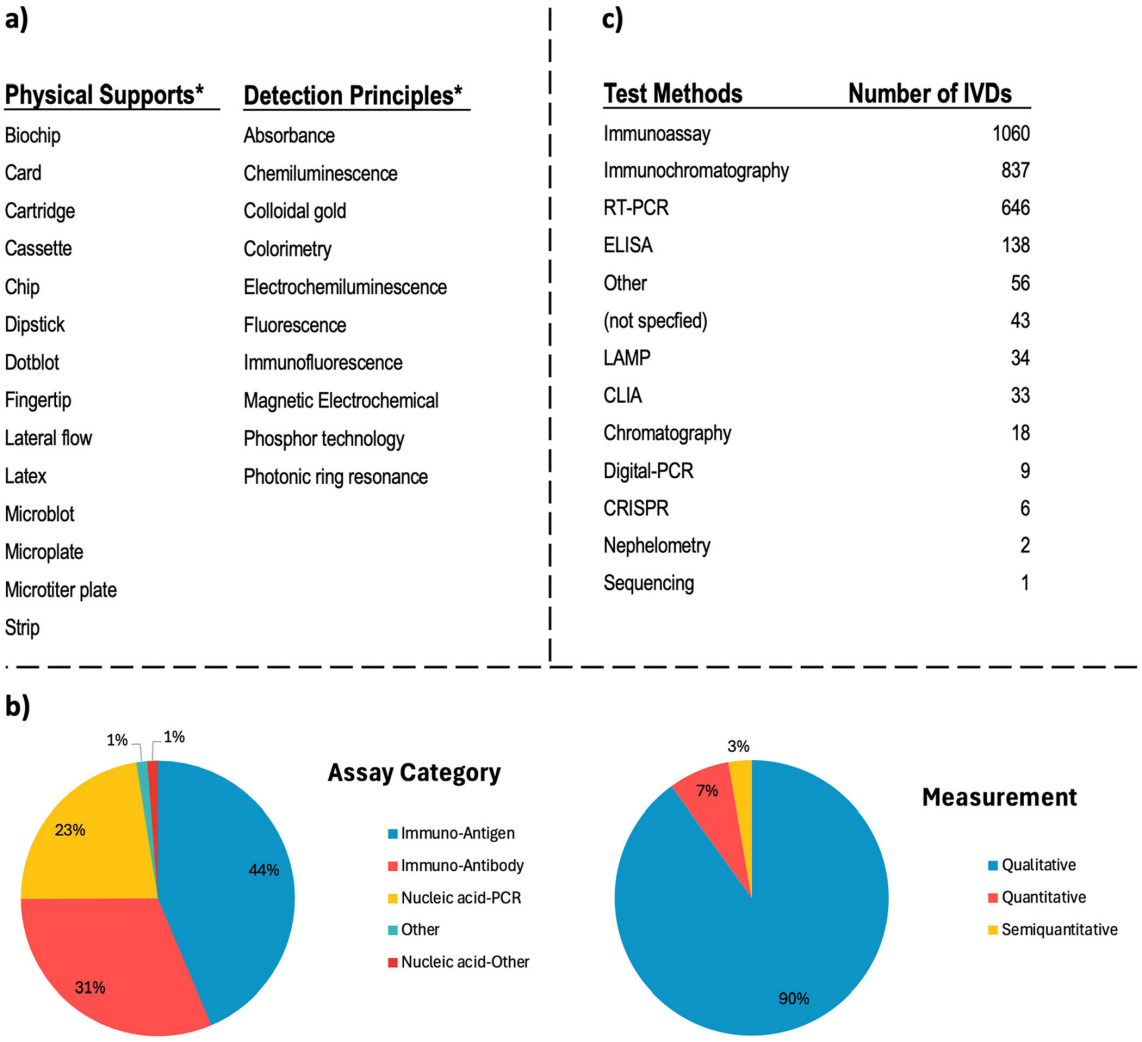
Distribution of IVDs and test kits in terms of technical choice. (a) Physical support types and detection principles of the products. (b) Distribution of products in terms of “assay category” and “measurement” types. (c) Distribution of products in terms of the test method technologies chosen. “*” represents the parameters whose total values could not be extracted from the data set.

IVD products developed for the detection of SARS‐CoV‐2 virus were developed using different physical supports and detection principles, but since there were not enough data entries in the database for analysis, only the physical supports and detection principles used are tabulated in Figure 3a. Despite the breadth of the data set analyzed, some limitations should be acknowledged. The reporting of technical details such as physical support materials and detection principles was missing across entries, restricting deeper comparative analysis of platform‐specific performance. Furthermore, the evaluation of detection limits (LOD) was complicated by the use of varied and sometimes manufacturer‐specific arbitrary units, particularly in immunoassay‐based products. This variability can limit standardization and benchmarking efforts across different assay types. Recognizing these limitations is also essential for not only for interpreting the findings of this study but also for national policies since they reduce the ability of decision‐makers to take data‐driven action on which materials and technologies, trained personnel, research projects and national/international supply chains are needed to ensure the security of the national/international supply chain for possible future pandemics.

When the data under the “assay category” heading, which groups the type of molecule targeted by IVD devices and test kits to detect the presence of SARS‐CoV‐2 virus, were analyzed, five different classifications emerged; immuno‐antigen (virus protein), immuno‐antibody (immunoglobulin), nucleic acid‐PCR (virus genome), nucleic acid‐other (virus genome), other (not specified) (Figure 3b). Looking at the distribution of IVD devices and test kits according to this classification, it was observed that antigen kits ranked first with 44%. Antibody kits, which can detect those who are sick and/or have been sick, accounted for 31% of the products, while PCR‐based products ranked third with 23%. In addition to commercial kits, PCR‐based in‐house laboratory experiments have also been used in the diagnosis of COVID‐19, which is the gold standard in virus detection, but it seems to cause a low diversity of commercial kits.

In terms of measurement, devices and kits that provide qualitative results were found to have an extremely dominant rate of 90%. When the “test methods” data, which can be positioned as a subclass of the assay category, is examined ‐ as expected – protein detection‐based test methods (1897 IVD devices and test kits in total), which can be used as POC, which are necessary for the management of the normalization process in health systems after strict lockdowns, are more preferred (Figure 3c). The fact that alternative methods (such as LAMP or CLIA) to “Immuno‐, PCR and ELISA” test methods, which can also be defined as conventional in diagnostic kits, are also used in commercial products, albeit at a low rate, is important in terms of providing an alternative to existing methods when developing strategies against a different outbreak in the future. Since we can only predict the parameters of the next “challenge” today, having methods with different advantages/disadvantages in our arsenal may create unexpected opportunities in the future.

**Figure 4:**
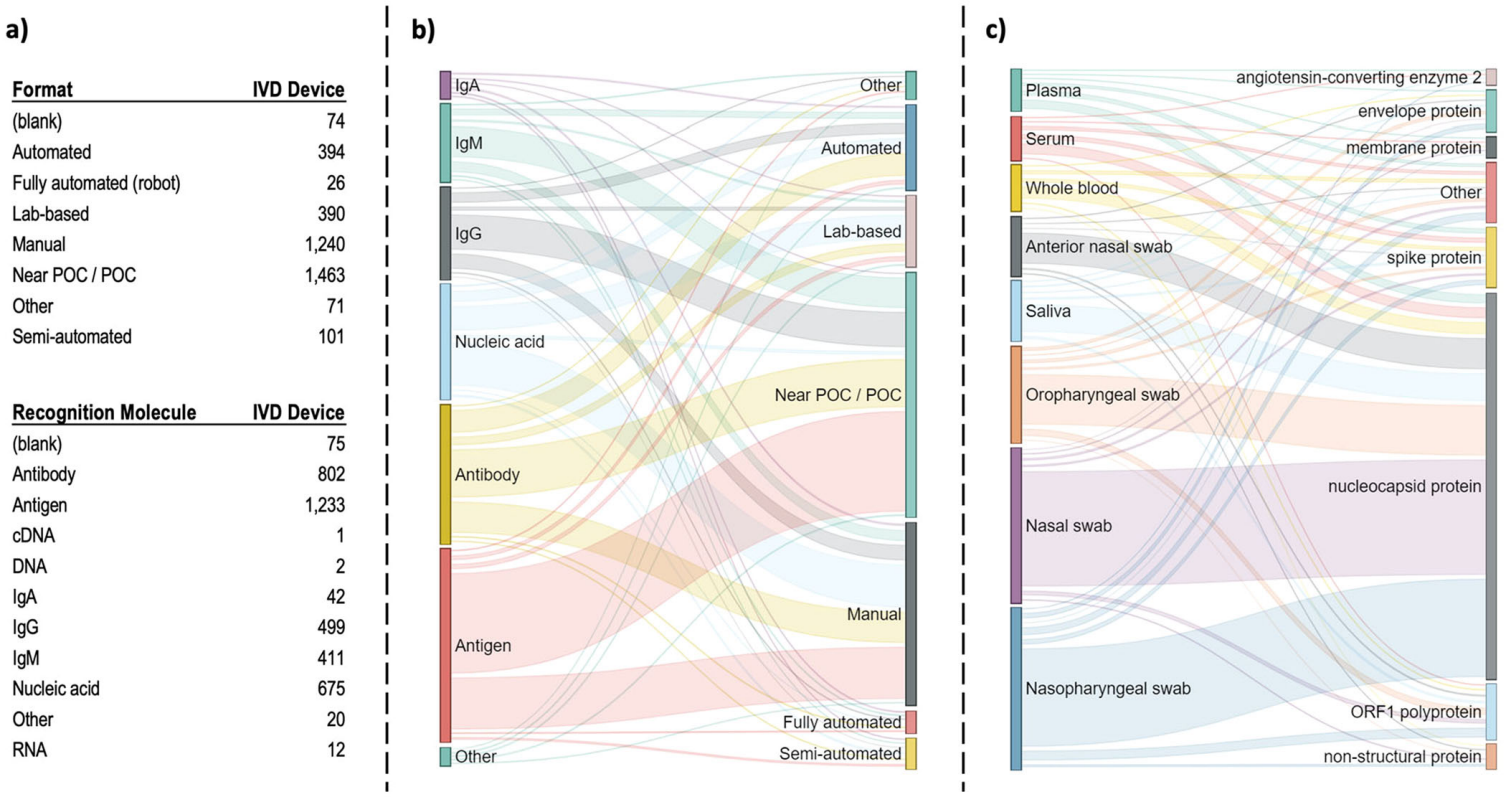
Distribution of strategies for consequtive challenges in commercial use; test format, recognition molecule, sample location, target molecule. (a) Number of different formats and recognition molecule chosen. (b) Flow diagram of products for matching between these two challenges. (c) Flow diagram of products matching the sample location and target molecule.

The results of the analysis for the development of COVID‐19 kits are summarized in Figure 4. There are basically four different decision points for a product to be developed for commercial use against COVID‐19: the format and detection molecule to be used are product‐related, while the sampling location and target molecule are sample‐related challenge points. In terms of format, seven different parameters were identified in the data set (Figure 4a) (Near POC/POC and Manual being the most frequently preferred), and it was found that the products followed different paths in the recognition molecule, sample location and target molecule parameters during the technical and commercial R&D processes (Figure 4b,c). Such strategic choices will vary depending on the nature of any disease agent, its symptoms and its location in the body. In the COVID‐19 pandemic, the advantage was that sampling was relatively easy, but we can see from the kits in this database that any course can be taken in the face of the challenge to biotech products posed by the expected future Disease X.

**Figure 5:**
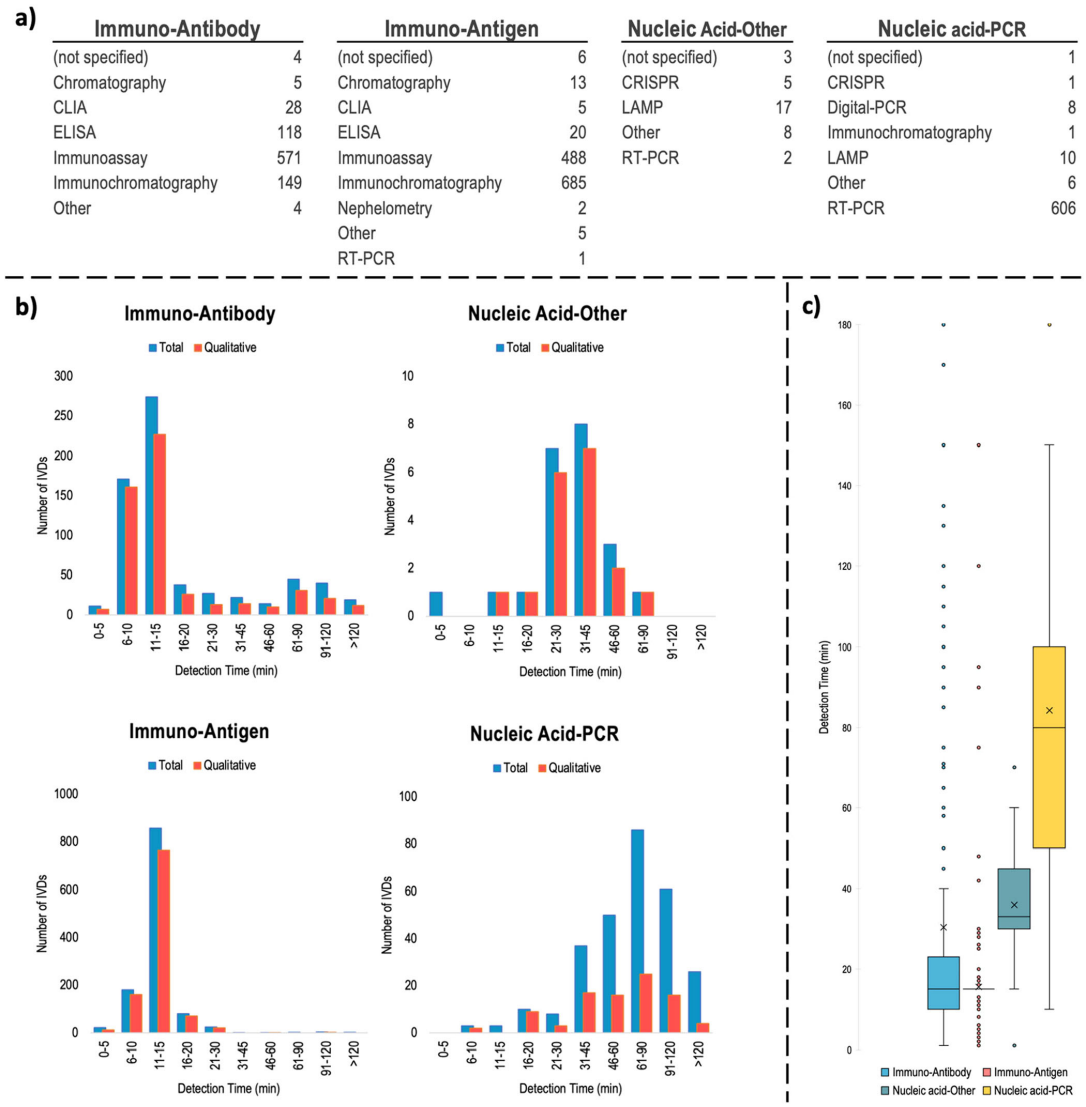
Detection time of the IVDs and test kits based on assay category, detailed in (a) Types of technologies used in different assay platforms. (b) Histogram distribution of detection times (for total and only qualitative products of the relevant assay category) in minutes. (c) Box‐whisker representation of detection limits based on assay category. Since 15 min is the extreme majority, the “box” of the Immuno‐Antigen graph has been observed as just a line.

The final focus for the IVD devices and test kits subject to the study was product validation, which is the most critical issue for their end‐use and commercial performance. As a result of the “assay category” based review, which we consider to be the most suitable parameter of the data set for analysis, the distribution of test methods for each assay category was determined (Figure 5a). Regardless of the test method used, the first parameter examined in the validation performances was “detection time”, which is also an important parameter of the decision mechanism during the COVID‐19 pandemic. As visualized in the histogram based on “assay category” in Figure 5b, although there is a dispersion in detection times due to the test method used, a clustering of each assay preferred based on “assay category” was identified. Antibody (a wider variance) and Antigen (a narrower variance) based test methods tend to give results within 15 min, whereas for antibody‐based tests, which are not POC, but rather lab‐based (and quantitative), this time spread up to 2 h (data not shown).

In the case of nucleic acid‐based detection methods, it was observed that the time distribution of the gold standard PCR technique was predominantly clustered in the 61–90 min band, whereas other nucleic acid‐based methods were clustered in the 21–45 min band. Technological breakthroughs to reduce the detection time of nucleic acid‐based detection methods may be vital for the possible future Disease X, as they provide much clearer results. A comparative analysis of the detection time distributions of different assay categories as a box‐whisker plot (Figure 5c) revealed that antigen‐based test kits showed the narrowest time distribution, while PCR‐based kits showed the widest time distribution (also related with sample preparation times). In addition to the need for skilled technicians and sophisticated equipment, the detection time and its variance among different producers/protocols will create new challenges against Disease X in the future about society mobilization control and engineering control measures during the pandemics.

**Figure 6:**
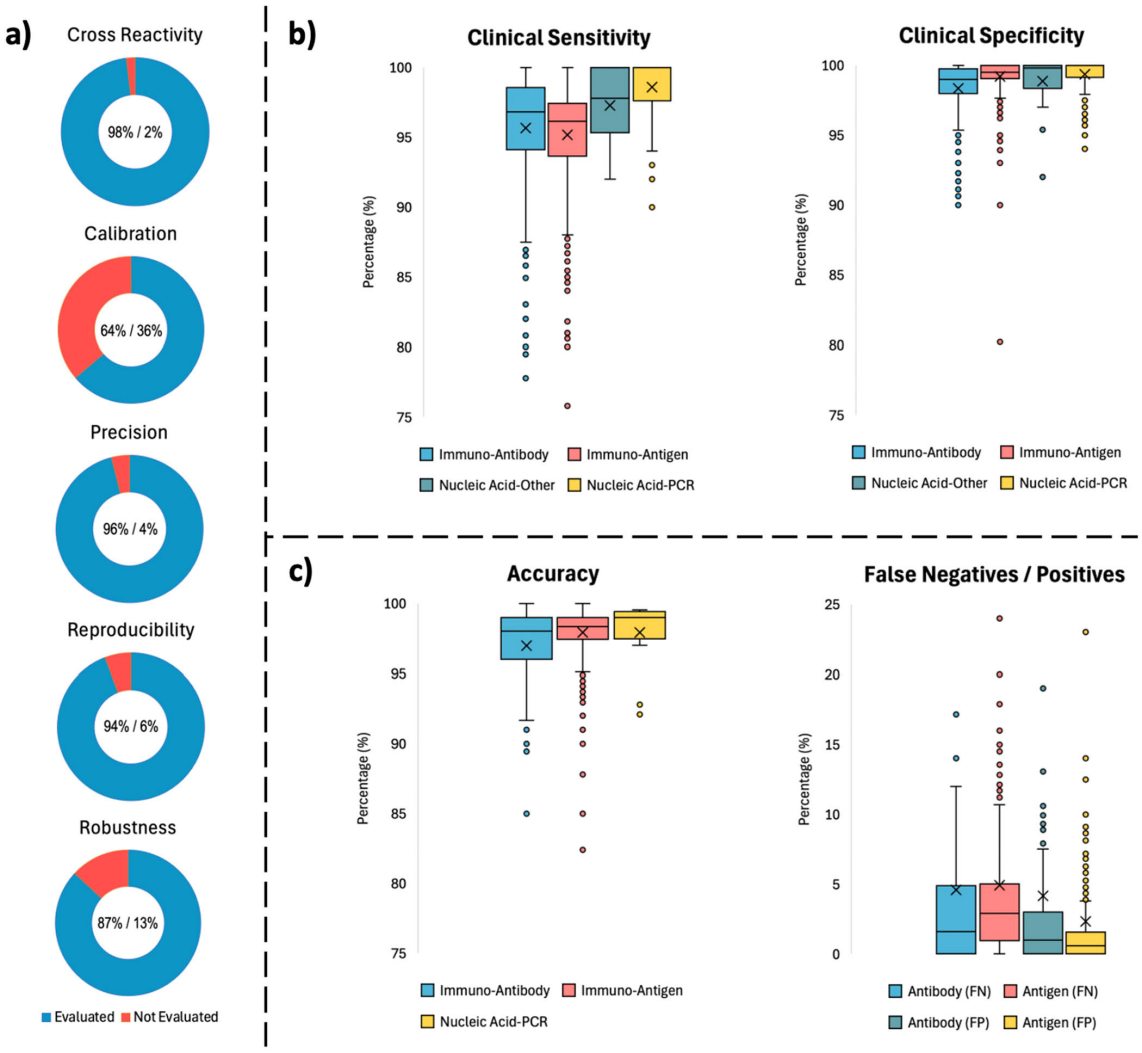
Validation parameters of IVDs and test kits. (a) Parameters being evaluated or not (without mentioning the numerical data in the data set). (b) Clinical sensitivity and specificity of the products to express the real‐world application performance. (c) Accuracy and false negatives/positives of the products having values in the data set.

In the continuation of the study, the performance of the biotechnology products examined was analyzed through other validation parameters. Although numerical data could not be obtained for some of the parameters, it was determined that “cross reactivity” was achieved 98%, “calibration” 64%, “precision” 96%, “reproducibility” 94% and “robustness” 87% in the scope of the experiments carried out before their use in the EU market (Figure 6a). Since calibration cannot be performed for all biotechnology products examined due to the technologies used, and some samples undergo pre‐purification due to the technology chosen, this observed low ratio can be interpreted. However robustness tests were performed relatively less in total of IVDs which may be crucial during suboptimal conditions of real‐life would challenge the IVDs' performances. Among the other validation parameters for which quantitative data were identified, “clinical sensitivity” and “clinical specificity” data are summarized in the graphs in Figure 6b to express the on‐site performance of the products. On the basis of “assay category,” it was found that the products in the Nucleic Acid‐PCR group, which is also accepted as the gold standard, had the highest clinical sensitivity on average (98.6% ± 2.0%). These products were followed by Nucleic Acid‐Other (97.3% ± 2.5%), Immuno‐Antibody (95.7% ± 4.4%), and Immuno‐Antigen (95.2% ± 4.3%). Within the scope of the clinical specificity parameter, the performance ranking was as follows: Nucleic‐Acid PCR (99.3% ± 1.2%), Immuno‐Antigen (99.2% ± 3.3%), Nucleic Acid‐Other (98.9% ± 2.0%), Immuno‐Antibody (98.3% ± 1.9%) group biotechnological products.

Since the data records for the products in the “Nucleic Acid‐Other” classification did not contain enough data to analyze for the “Accuracy” parameter, the remaining three classifications were analyzed; the average accuracy of the products in the Nucleic Acid‐PCR and Immuno‐Antigen classes were close to each other (97.9% ± 2.5% and 97.9% ± 3.4%, respectively), while the accuracy of the Immuno‐Antibody class products was 96.6% ± 5.3% (Figure 6c). “False Negatives/Positives” are only available for antibody and antigen test kits in the data set. When false positive (FP) and false negative (FN) values were averaged, the FP rate of antigen kits showed better results than antibody kits (2.3%–4.1%), while for the FN rate, antibody kits slightly outperformed antigen kits (4.6% to 4.9%). The false negative rate, which is a challenge to be considered in controlling pandemics, is a point where R&D studies will be a challenge point in the future.

The validation parameters mentioned in Figure 6 are crucial for national/regional approval of the IVDs for mass‐scale use by health professionals and patients and in the event of such a global crisis, balancing speed with rigorous validation was as challenging as dealing with the virus itself. During the COVID‐19 pandemic, countries adopted a variety of emergency regulatory mechanisms to accelerate the availability of diagnostic tests. In the United States, the FDA implemented the Emergency Use Authorization (EUA) mechanism to accelerate the availability of in vitro diagnostics (IVDs) (FDA 2023a). In China, the National Medical Products Administration (NMPA) activated a “green channel” that enabled conditional approval of diagnostics based on limited initial data, with post‐market performance evaluation expected to follow (NMPA 2024). Similarly, Japan employed its “Special Approval for Emergency” (SAE) pathway, allowing for temporary authorization of tests when no existing alternatives were available and public health was at risk (Maeda 2021). In South Korea, the Ministry of Food and Drug Safety (MFDS) rapidly authorized COVID‐19 diagnostics through an Emergency Use Authorization (EUA) system modeled after the U.S. FDA, with national reference laboratories verifying product performance within days (MFDS 2020). One of the lessons learned from this pandemic is the need for internationally aligned protocols in future pandemic responses. Thus, the criteria for sensitivity, specificity, and limit of detection (LOD) should be harmonized to compare diagnostics across regions. Also, robust post‐market surveillance frameworks should be put in place, especially with diverse clinical and demographic settings.

**Figure 7:**
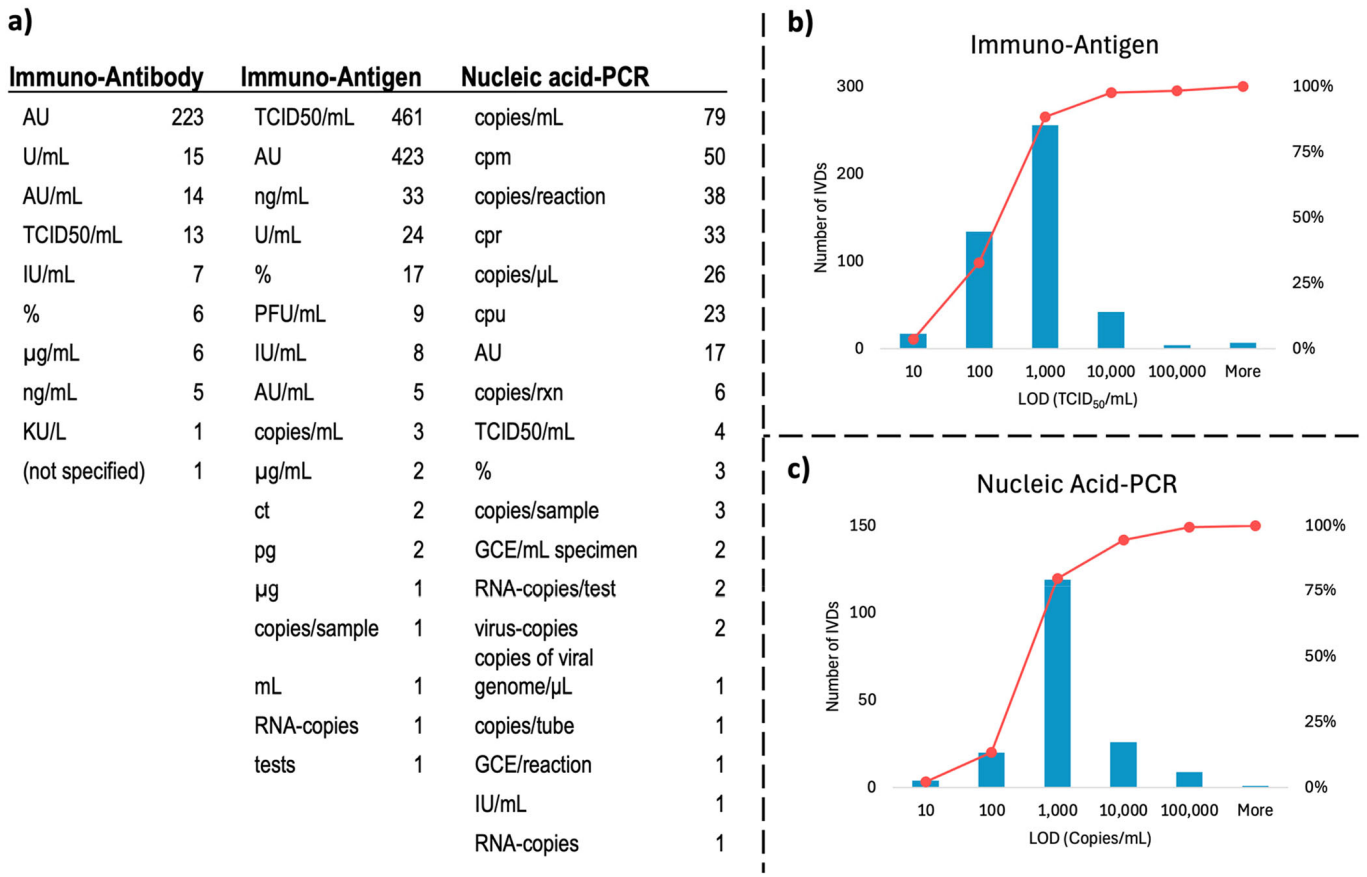
Limit of detection parameters of IVDs and test kits. (a) Units of LODs for different assay categories. For the sake of tranparency, the original data of units from the data set were noted without any change. (b) Histogram of LOD distribution for Immuno‐Antigen test kits. (c) Histogram of LOD distribution for Nucleic Acid‐PCR test kits. Red lines in (b) and (c) represent the cumulative percentage of the distribution.

The last parameter examined within the scope of validation results and performance of IVD devices and test kits was the “limit of detection” (LOD). When product performances were analyzed for “assay category” classification (three different categories having enough data), different units of measurement were encountered (also due to the test methods used). Another difficulty encountered was the presentation of “arbitrary unit (AU)” units in antibody and antigen‐based products, which is a definition of the manufacturer that emerged as a result of the manufacturer's optimization efforts (Figure 7a). In this context, “TCID_50_/mL” (Median Tissue Culture Infectious Dose) for antigen‐based kits and “copies/mL” for PCR‐based kits were used as comparable parameters (including other units of PCR kits that can be converted into copies/mL). When detection limits are analyzed as a semi‐logarithmic graph, 88.5% of antigen‐based products are positive at 1000 TCID_50_/mL and above, compared to 80.0% of PCR‐based products at 1000 copies/mL and above (Figure 7b,c). A study with COVID‐19 kits found that each 10‐fold increase in detection limits increased the false negative rate by 13% (Arnaout et al. 2020). In this context, since it is a parameter that directly determines false negative rates, the extent to which detection limits should be technically improved in the fight against future epidemics can be determined with these average figures of products in the market.

The COVID‐19 pandemic underscored the importance of rapid, scalable testing methods and adaptive national policies in epidemic control. What we did not have during COVID‐19 pandemic will be available and most probably have a much bigger effect on humanity in the future than the pandemic itself, artificial intelligence. For future outbreaks, integrating big data management and AI‐assisted surveillance with diagnostic infrastructure will be vital for real‐time monitoring, early detection, and predictive modeling of disease spread. Point‐of‐care testing must be complemented by national strategies that ensure equitable access and rapid regulatory responses. There will be a need for harmonized data systems, agile governance, and innovation in diagnostics to effectively control the development of epidemics in the event of a resurgence (Shilo et al. 2020; Topol 2019). It will be vital in the event of upcoming infectious disease to track the spread and potential (location, population, symptoms and mutation‐based) paths of pathogen on an ongoing‐basis via integrating different diagnostics results (which will most probably be crypted data packages of patient, location, date, sampling method, technologies used, test results, etc.) accustomed to be analyzed for flexible and smart emergency management. This will lead us to the harmonization capability of diagnostics data from different technologies. A “yes/no” for the status of patient will maybe somewhat less sophisticated, but a direct approach in managing the spread of a disease. However, any capability to concatenate different test results and other health‐related data in a comparative and analyzable way (like socio‐environmental factors, medical history data, pathogen load, current health parameters, standardized/classified symptoms) will catalyze the understanding of the dynamic nature of infectious disease as a public health problem.

## 4 Conclusion

In summary, we have reported herein the technical preferences, limitations, and other performance parameters of IVDs and test kits that could be developed against a future Disease X. Two different data sets, “The Web of Science (WoS) database” and “JRC COVID‐19 IVD Devices and Test Methods” data sets, were used for analysis. According to the number of research articles on a country basis, the United States and China are found as the primary developers and of diagnostic kits. In five different classifications based on the type of molecule targeted by IVD devices and test kits to detect the presence of the SARS‐CoV‐2 virus, antigen kits ranked first with 44%. When the distribution of products in terms of test method technologies chosen was examined, protein detection‐based test methods were used in 1897 IVD devices and test kits in total. Test format, recognition molecule, sample location, and target molecule are decision points for a product to be developed for commercial use. Near POC/POC and Manual was the most frequently preferred test format for COVID‐19 kits. It is observed that the antigen was used as a recognition molecule in 1233 IVD devices. In terms of sample location and target molecule parameters, IVD kits followed different paths. Detection time is the first parameter examined in the validation performances. There is a dispersion in detection times up to 2 h due to the test method used. Data of “clinical sensitivity” and “clinical specificity” were identified data as other validation parameters to express the on‐site performance of the products. Within the scope of the clinical sensitivity and clinical specificity parameters, the products in the Nucleic‐Acid PCR was found as 98.6 ± 2.0% and 99.3 ± 1.2% on average, respectively. The average accuracy of the products in the Nucleic Acid‐PCR and Immuno‐Antigen classes was found to be close to each other. “False Negatives/Positives” are only available for antibody and antigen test kits in the data set. The FP rate of antigen kits showed better results. “Limit of detection” (LOD) was the last parameter for validation results and performance of IVD devices and test kits, and different units of measurement were encountered. According to detection limit analyses as a semi‐logarithmic graph, it was found that 88.5% of antigen‐based products are positive at 1000 TCID_50_/mL and above, compared to 80.0% of PCR‐based products at 1000 copies/mL and above. In conclusion, this review highlights the global efforts and innovations in diagnostic technologies during the COVID‐19 pandemic, focusing on In Vitro Diagnostics (IVDs). The analysis of 2882 biotechnological products provides valuable insights into test formats, validation performances, and detection limits, emphasizing the dominance of antigen‐based kits and the high accuracy of PCR‐based methods. Key decision points, such as sampling locations, recognition molecules, and target molecules, are discussed in the context of pandemic preparedness. This study serves as an important resource for guiding future developments against Disease X by identifying technological gaps and opportunities for rapid and effective diagnostics.

Based on the findings of this study, the authors suggest areas for improving diagnostic preparedness in anticipation of future pandemics. First, the standardization of validation metrics—such as sensitivity, specificity, and limit of detection—should be prioritized to ensure the comparability and reliability of diagnostic performance across platforms and regions, which will be more crucial as geopolitical factors pose increasing risks to the fluidity and resilience of future supply and trade chains. Second, greater regulatory alignment and cross‐border collaboration are needed to harmonize approval processes and facilitate global access to validated diagnostics during health emergencies. Facing a so‐called “small scale enemy” irrelevant and independent from “borders” and “differences”, will compel “large scale standardization” for approval of diagnostics in a safe clinical application in a so‐called “battlefield”. Finally, retrospectively, post‐market surveillance should be conducted based on what we have learned from COVID‐19 for on‐field performance of IFDs to address two challenges; what will be the preferable technologies in terms of robust field performance and, what will be the generalized post‐market surveillance system for emergency use frameworks to monitor real‐world diagnostic performance and ensure sustained accuracy. Collectively, these recommendations underscore the importance of linking technological innovation with regulatory foresight and data infrastructure to strengthen pandemic response capacity.

## Author Contributions

**Murat Kavruk:** conceptualization, writing – original draft, writing – review and editing. **Meltem Ercan:** conceptualization, writing – review and editing. **Baris Ata Borsa:** conceptualization, writing – review and editing. **Veli Cengiz Özalp:** conceptualization, writing – review and editing. **Frank J. Hernandez:** conceptualization, writing – review and editing.

## Ethics Statement

The authors have nothing to report.

## Conflicts of Interest

The authors declare no conflicts of interest.

## Data Availability

Data sharing is not applicable to this article as no data sets were generated or analyzed during the current study.
